# Differences in muscle activity and intermuscular coordination between dominant and non-dominant hands during chopstick manipulation

**DOI:** 10.3389/fnhum.2025.1574002

**Published:** 2025-04-09

**Authors:** Hina Komi, Hiroshi Kurumadani, Kazuya Kurauchi, Shota Date, Toru Sunagawa

**Affiliations:** Laboratory of Analysis and Control of Upper Extremity Function, Graduate School of Biomedical & Health Sciences, Hiroshima University, Hiroshima, Japan

**Keywords:** intermuscular coordination, muscle activity, chopstick manipulation, muscle synergy, dominant hand, non-dominant hand

## Abstract

**Introduction:**

To develop an efficient rehabilitation program for patients with stroke to acquire fine motor skills such as chopstick manipulation, it is necessary to examine the differences in fundamental muscle functions between the hands during motor tasks. The aim of this study was to clarify the differences in muscle activity and intermuscular coordination between dominant and non-dominant hands during chopstick manipulation.

**Methods:**

Twenty-eight healthy adults performed the task of picking up different-sized objects using chopsticks with either their dominant or non-dominant hand. Surface electromyography of 11 intrinsic and extrinsic hand muscles was performed, and muscle activity and muscle activity waveforms during the task were calculated. Activity patterns and weighting for each pattern were extracted from the muscle activity waveforms using non-negative matrix factorization to represent muscle synergy. The muscle activity and weighting were compared between the dominant and non-dominant hands and among different-sized objects.

**Results:**

The activities of most intrinsic and extrinsic muscles did not significantly differ between the dominant and non-dominant hands or among different-sized objects. Although activity patterns showed the coordination of intrinsic hand muscles in both the dominant and non-dominant hands, the combinations of the weighting differed between the dominant and non-dominant hands. The non-dominant hand had different muscle activation patterns of intrinsic and extrinsic hand muscles compared to the dominant hand. The activity patterns and weighting were mostly similar across different-sized objects.

**Conclusion:**

The dominant hand showed coordination of the first and second lumbrical muscles, whereas the non-dominant hand showed no muscle activation patterns between the muscles. Therefore, it is important to emphasize the first and second lumbrical in the non-dominant hand during rehabilitation to improve the coordination between the muscles of the two hands during chopstick manipulation to effectively improve chopstick manipulation skills in the non-dominant hand.

## 1 Introduction

Paralysis of the dominant upper limb due to stroke or other neurological conditions can severely impair daily activities and substantially reduce quality of life, as individuals primarily rely on their dominant hand for tool manipulation (Langhorne et al., 2009). A rehabilitation approach for the paralyzed upper limb is “dominant hand exchange,” wherein movements previously performed with the dominant hand are trained using the non-dominant hand. This approach typically requires extensive repetitive training, with particularly intensive effort needed for acquiring complex fine motor tasks (Dayan and Cohen, 2011). One such complex fine motor task is chopstick manipulation. Using chopsticks is an essential daily activity, especially in East Asian countries (Lee and Chen, 2008), and therefore, there is a demand for reacquiring chopstick manipulation skills through dominant hand exchange rehabilitation. Previous research has demonstrated that acquiring chopstick manipulation with the non-dominant hand requires long-term training, comparable to other skill reacquisition processes in dominant hand exchange (Sawamura et al., 2019; Park and Son, 2022). To achieve acquisition of these skills, including chopstick manipulation, with non-dominant hand through shorter-term training, it is crucial to identify the fundamental functions of muscles involved in these motor tasks.

Previous studies on chopstick manipulation with the dominant hand have shown that both intrinsic and extrinsic hand muscles are involved in a coordinated manner (Kurauchi et al., 2024). However, studies on muscle activity and intermuscular coordination during chopstick manipulation in the non-dominant hand are lacking, and the similarities and differences in the functions of muscles between the dominant and non-dominant hands remain unclear. One study has demonstrated that muscle activity differs between the dominant and non-dominant hands even when performing the same task. Nishizawa and Toriizuka (2019) reported that during tasks requiring precise force control, the non-dominant hand exhibited excessive muscle activity compared to the dominant hand. Although a few studies have compared intermuscular coordination between hands, muscle synergy analysis can reveal patterns of muscle cooperation. This method determines common temporal patterns among multiple muscles and quantifies their respective contributions (Merkle et al., 1998), enabling detailed comparisons between limbs.

The aim of this study was to clarify the differences in muscle activity and intermuscular coordination during chopstick manipulation between the dominant and non-dominant hands. We tested the hypothesis that chopstick manipulation with the non-dominant hand can result in excessive muscle activity and distinct intermuscular coordination compared with those of the dominant hand. The findings of this study are expected to provide valuable insights for developing efficient rehabilitation strategies to train the non-dominant hand for chopstick use in patients with stroke.

## 2 Materials and methods

### 2.1 Participants

Twenty-eight right-handed adults participated in this study. None of the participants had any hand disability. All participants were accustomed to using chopsticks on a daily basis. The sample size was determined using *a priori* power analysis with G*Power statistical packages (G*Power Ver. 3.1.9.7; Universität Düsseldorf, Düsseldorf, Germany), based on a three-way independent measures analysis of variance (ANOVA; fixed effects, special, main effects, and interactions). This analysis yielded a sample size of 26 participants (effect size = 0.6; *α* = 0.05; power = 0.8; Number of groups = 12). To account for potential data loss due to measurement errors, participant exclusions, or missing data, we recruited 28 participants to ensure the robustness of the statistical analysis and maintain a sufficient sample size, even if some data were excluded. Participants were divided into two groups using stratified randomization: one group consisted of 14 participants (eight male and six female individuals; mean age 23.7 ± 3.9 year) who performed the tasks with their dominant hand and the other group consisted of 14 participants (seven male and seven female individuals; mean age 21.6 ± 1.4 year) who performed the tasks with their non-dominant hand. Informed consent was obtained from all participants.

### 2.2 Experimental tasks

**Figure 1:**
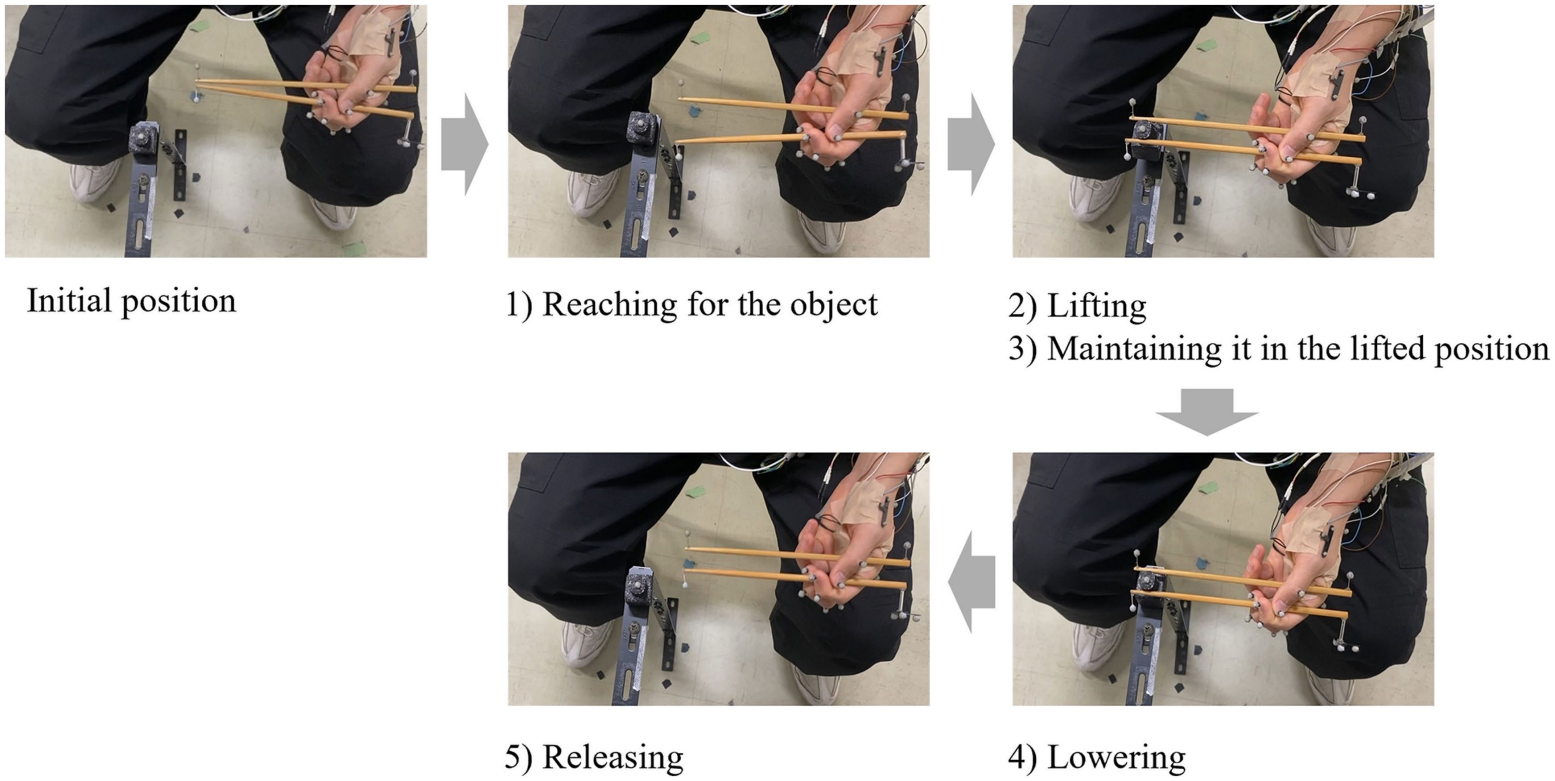
Procedure of the experimental task.

We used a 225-mm-long, 10.3-g bamboo chopstick with a square-shaped handle in the experiment. Each participant was made to sit on a chair. Both elbow joints were slightly flexed. The participants held the chopsticks in the non-tasking hand as the initial limb position. On cue, the chopsticks were switched to the hand performing the task, and five movements were performed with the chopsticks: (1) reaching for the object, (2) grasping and lifting the object, (3) maintaining the lifted position, (4) lowering, and (5) releasing the object (Figure 1). The object to be grasped was a cylindrical foam object weighing 10 g and of varying sizes (1, 2, and 3 cm diameter), resulting in three conditions. The task conditions were randomized. Each action was synchronized for 1 s, with a metronome set at 60 beats/min. Twelve trials were conducted under each condition.

### 2.3 Measurements

#### 2.3.1 Muscle activity

**Figure 2:**
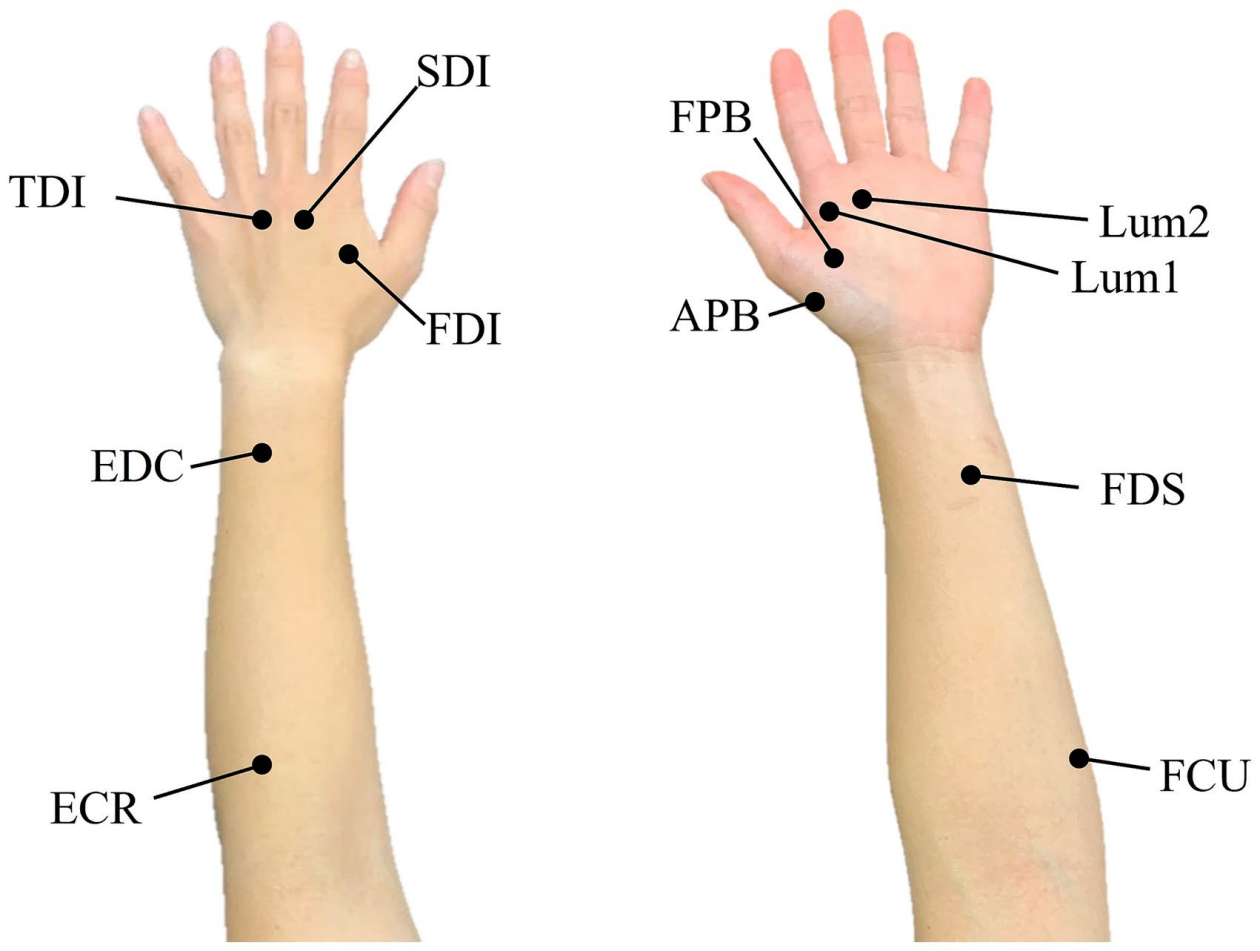
Position of electrodes: the left and right panels show the dorsal and palmar side, respectively.

The activity of 11 muscles was measured, including seven intrinsic and four extrinsic hand muscles, during object grasping with chopsticks. These muscles included the first lumbrical muscle (Lum1), second lumbrical muscle (Lum2), abductor pollicis brevis (APB), flexor pollicis brevis (FPB), first dorsal interosseous muscle (FDI), second dorsal interosseous muscle (SDI), third dorsal interosseous muscle (TDI), flexor digitorum superficialis (FDS), extensor digitorum muscle (EDC), flexor carpi ulnaris (FCU), and extensor carpi radialis (ECR) (Figure 2). The measurements were performed using a wireless electromyograph (Intercross-413; Intercross Inc., Tokyo, Japan).

In the electromyography (EMG) setup, the muscle belly positions of each muscle were identified using an ultrasound device (SONIMAGE MX1; Konica Minolta, Tokyo, Japan). Marks were placed on the muscle belly positions using an oil-based pen. An ultrasound device was used to place a cotton thread directly above the muscle belly of the lumbrical muscles to confirm the location, and the attachment sites were determined accordingly (Kurumadani et al., 2023), and surface electrodes were placed on the muscles. Second, the skin surface around the target muscles was prepped by polishing with a skin pretreatment gel for bio-signal monitoring (SkinPure; Nihon Kohden, Tokyo, Japan), followed by cleansing with alcohol-soaked cotton. Lastly, disc electrodes with a diameter of 3 mm were attached to Lum1 and Lum2 and disc electrodes with a diameter of 8 mm were attached to the other muscles. A paste for the EMG was applied, and the electrodes were affixed to the skin. The electrode distance was 7 mm for the 3 mm electrodes and 15 mm for the 8 mm electrodes. Generally, the distance between electrodes is 10–20 mm; however, when the muscle cross-sectional area is small, it is necessary to use small surface electrodes and a narrower distance between electrodes (Winter et al., 1994). Muscle activity during the task was recorded at a sampling frequency of 1,000 Hz.

#### 2.3.2 Chopstick distance

To track the movement of the chopsticks during the task, we attached superficial reflective markers to the chopstick tips. The positional coordinates of these markers were determined using Motive software (OptiTrack; Acuity Inc., Tokyo, Japan) in conjunction with 16 infrared cameras (Flex3; Acuity Inc., Tokyo, Japan). The positional coordinates of the markers were recorded at a sampling frequency of 100 Hz. Chopstick movement data were electrically synchronized with the EMG data.

### 2.4 Experimental procedure

The participants were instructed how to perform the grasping task and were allowed to practice until they could adequately perform the task. Surface electrodes and reflective markers were attached (as described above), and the task was performed using either the dominant or non-dominant hand. The object was presented in three sizes: 1, 2, and 3 cm, and the order of the object size was randomized. Twelve trials were conducted for each object. After the task, maximum voluntary contraction (MVC) of each muscle was measured.

### 2.5 Analytical approach

In this study, muscle activity waveforms and intrinsic and extrinsic hand muscle activity during chopstick manipulation were analyzed using MATLAB R2023b (MathWorks, Massachusetts, USA).

#### 2.5.1 Section to be analyzed

**Figure 3:**
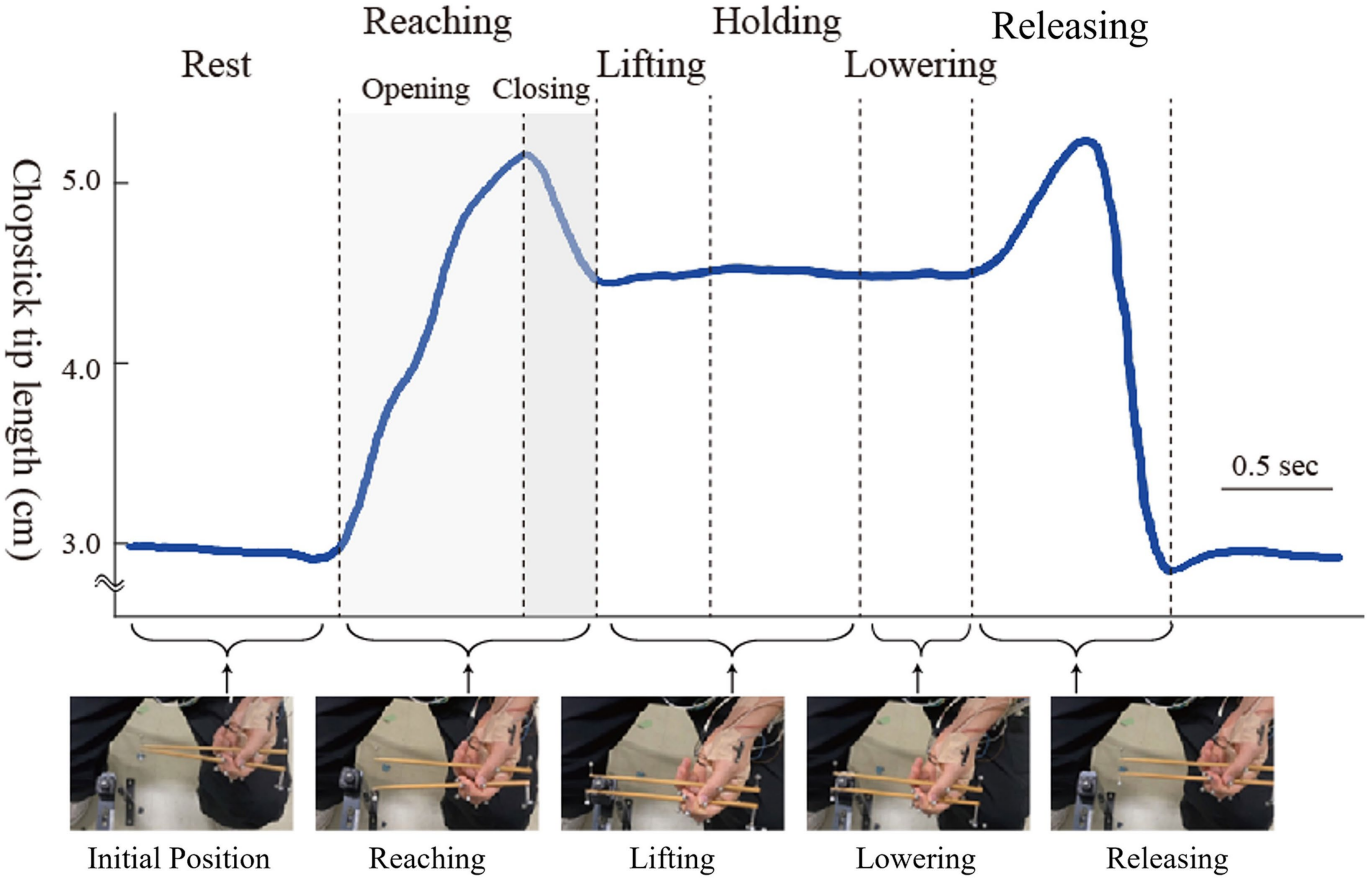
Analysis of the target section: the blue line represents the distance between the chopstick tips obtained from the marker coordinates.

The interval to be analyzed was from the start of opening and closing the chopsticks to grasping the object. As we focused on the opening and closing of the chopsticks, we excluded from the analysis the sections involving the lifting and lowering of the object that did not involve any obvious movement of the chopsticks. The distance between the chopstick tips was determined using marker coordinate data. Subsequently, three key points were identified: the start of chopstick opening, maximum opening, and object grasping. These points were categorized into two phases: opening and closing (Figure 3).

#### 2.5.2 Analysis

The obtained muscle activity waveforms were filtered using a bandpass filter (cutoff frequency of 20–450 Hz). Subsequently, the root mean square (RMS) for a duration of 1 s was computed, and the MVC ratio was derived from the RMS value for each muscle. The average of five consistent trials for each task was used as a representative value for each participant.

For each participant, non-negative matrix factorization was applied to the complete muscle activity data to calculate the time-varying patterns and muscle weights (Lee and Seung, 1999). The optimal number of synergies was determined based on the Variance Accounted For (VAF), which measures the extent to which the data reconstructed by the NMF reproduces the original data. The VAF was calculated starting with one synergy, and the optimal number of synergies was defined as the point where the VAF exceeded 90% and the increase in VAF was <5% with additional synergies (Yokoyama et al., 2021). In each synergy, muscle weighting components are expressed on a scale from 0 to 1, with higher values indicating a greater contribution to the synergy. In this study, a muscle weighting component of 0.4 or higher was considered indicative of active coordination within that synergy (McGowan et al., 2010). Scalar product (SP) is used to evaluate the similarity of muscle synergies (Saito et al., 2018); the SP values range from 0, indicating no similarity in waveform, to 1, indicating complete similarity in waveform. In this study, an SP value >0.8 was defined as indicating a high degree of similarity (Oliveira et al., 2013). Muscle synergy values obtained for the 1-cm object size condition were used as a reference, and calculations were performed for all combinations.

### 2.6 Statistical analysis

The effects of hand dominance on the intrinsic and extrinsic hand muscle activities during chopstick manipulation were analyzed for each muscle using a three-way repeated measures analysis of variance (ANOVA) with chopstick phase (opening and closing), hand dominance (dominant, non-dominant), and object size (1, 2, and 3 cm) as factors in each muscle. The Bonferroni test was used for *post-hoc* comparisons to determine differences in the effects of object size if the ANOVA results were significant. All statistical analyses were performed using R version 4.3.1. The level of significance was set at *p* < 0.05.

## 3 Results

### 3.1 Muscle activity

**Figure 4:**
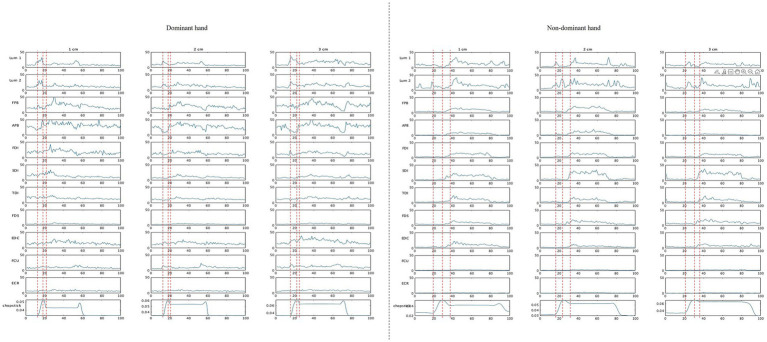
Representative muscle activity waveforms. APB, abductor pollicis brevis; ECR, extensor carpi radialis; EDC, extensor digitorum communis; FCU, flexor carpi ulnaris; FDI/SDI/TDI, first/s/third dorsal interossei; FDS, flexor digitorum superficialis; FPB, flexor pollicis brevis; Lum1/Lum2, first/s lumbrical muscle. The red dotted lines indicate the point at which the chopsticks begin to open, reach their maximum, and grasp an object, respectively.

In Figure 4, we have illustrated typical muscle activity waveforms during chopstick manipulation for each muscle. In the dominant hand, Lum1 and Lum2 tended to show increased muscle activity at the start of chopstick opening and closing. Additionally, the intrinsic hand muscles, including the FPB, APB, FDI, SDI, and TDI, tended to show increased activity during the closing phase. In the non-dominant hand, Lum1, Lum2, FPB, and APB tended to show increased activity during both opening and closing of the chopsticks. Table 1 shows the results of the three-way ANOVA for each muscle type. A significant main effect of the chopstick phase (opening and closing) was observed for all muscles except the FCU. Additionally, a significant main effect of hand dominance was found for the TDI and ECR (TDI: *p* < 0.01, *η*^2^ = 0.282, ECR: *p* < 0.05, *η*^2^ = 0.193). An interaction between the chopstick phase and hand dominance was noted for the FDS, ECR, and FCU. The *post-hoc* tests revealed that the activity of the FDS was significantly greater during the closing phase than during the opening phase for the non-dominant hand. In the FCU, the activity was significantly greater during closing than during opening for the dominant hand, and activity of the ECR was significantly greater during closing than during opening for both the dominant and non-dominant hands. Furthermore, the activity of the ECR was significantly greater in the non-dominant hand than in the dominant hand during both the opening and closing phases.

**Table 1:** Activities of each muscle in the non-dominant and dominant hands during chopstick opening and closing.

		Non-dominant hand			Dominant hand			Factor					
								Hand dominance		Chopstick phase		Width	
	Width	1 cm	2 cm	3 cm	1 cm	2 cm	3 cm	F	*P*	F	*P*	F	*P*
Muscle	Chopstick phase												
Lum1	Opening	22.6	23.5	23.5	16.9	16.3	16.9	1.4	0.3	12.4	<0.001	0.3	0.76
	Closing	28.1	28.0	28.3	21.9	25.6	24.1						
Lum2	Opening	22.7	17.4	18.0	17.0	15.7	17.7	2.5	0.1	22.7	<0.001	1.9	0.16
	Closing	29.1	30.7	32.3	23.4	24.7	26.9						
FPB	Opening	16.6	15.5	16.3	16.0	17.4	18.3	0.4	0.6	57.1	<0.001	0.2	0.86
	Closing	28.2	26.0	25.7	28.4	29.4	30.8						
APB	Opening	8.5	8.4	8.3	9.2	10.1	9.9	0.1	0.8	16.8	<0.001	0.6	0.58
	Closing	16.8	12.8	15.3	14.5	14.7	16.4						
FDI	Opening	13.9	15.2	17.8	9.4	9.7	9.9	3.0	0.1	18.3	<0.001	0.8	0.46
	Closing	18.2	18.6	19.2	13.7	13.6	13.7						
SDI	Opening	10.9	10.8	11.6	5.9	6.6	6.3	1.7	0.2	43.8	<0.001	0.0	0.98
	Closing	16.5	15.7	15.3	14.4	15.2	15.3						
TDI	Opening	18.1	20.2	19.4	8.5	10.0	9.3	10.2	<0.01	36.7	<0.001	0.5	0.57
	Closing	27.3	27.2	25.3	16.0	18.0	16.9						
FDS	Opening	11.4	9.5	10.1	6.0	6.6	7.6	2.5	0.1	17.9	<0.001	3.0	0.89
	Closing	14.5	15.0	11.8	6.9	7.4	7.8						
EDC	Opening	12.0	13.4	14.8	11.0	11.6	11.5	2.0	2.0	25.0	<0.001	2.3	0.11
	Closing	17.2	18.3	19.3	13.9	14.8	14.5						
FCU	Opening	6.2	5.9	6.7	6.5	7.8	7.9	0.0	1.0	0.03	0.86	1.5	0.24
	Closing	7.0	6.7	7.8	5.7	7.0	6.7						
ECR	Opening	8.4	10.2	9.5	6.2	6.2	6.3	6.2	<0.05	32.6	<0.001	1.6	0.22
	Closing	12.0	14.5	12.2	7.5	7.6	7.5						

APB, abductor pollicis brevis; FDI first dorsal interosseous muscle; FDS, flexor digitorum superficialis; FPB, flexor pollicis brevis; EDC, extensor digitorum communis; ECR, extensor carpi radialis; FCU, flexor carpi ulnaris; Lum1, first lumbrical; Lum2, second lumbrical; SDI, second dorsal interosseous; TDI, third dorsal interosseous muscle.

### 3.2 Muscle synergy

**Figure 5:**
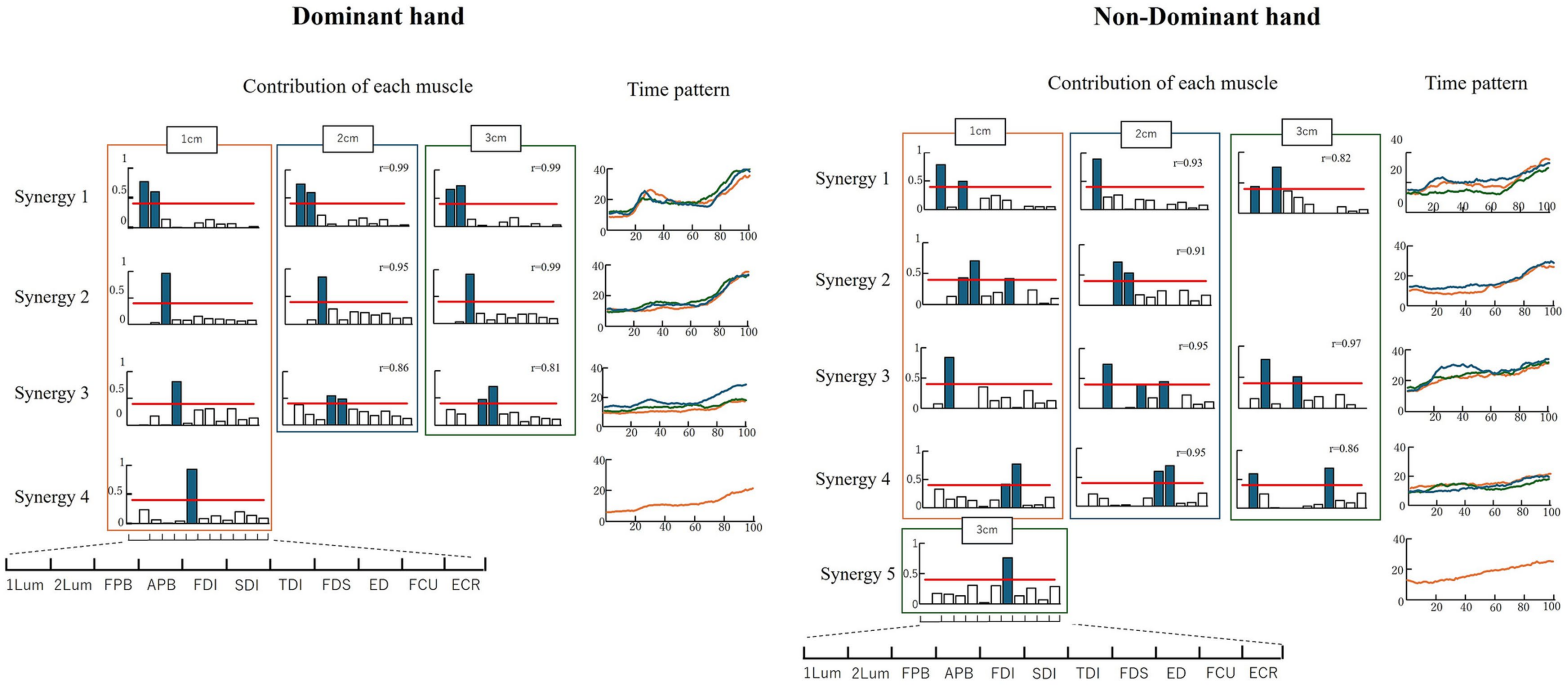
Results of the muscle synergy analysis. The red line indicates a value of 0.4. The colored bar graph shows a contribution ratio of 0.4 or more, indicating synergistic effect muscle. “r” is the similarity index.

Figure 5 shows the results of the analysis of muscle synergies. For the dominant hand, four muscle synergies were obtained at 1 cm and three at 2 and 3 cm. Classification using the SP method based on the synergies obtained at 1 cm yielded four synergies. Synergy 1 is a pattern of increased muscle activity in the opening–closing phase, indicating intermuscular coordination between Lum1 and Lum2. Synergies 2, 3, and 4 are patterns of increased muscle activity in the closing phase, with synergy 2 showing FPB activity and synergies 3 and 4 showing APB and FDI muscle coordination. For the non-dominant hand, four synergies were obtained for all object sizes. The SP method was used to classify the synergies into five types. Synergy 1 was a pattern of increased muscle activity with the opening–closing phase, showing intermuscular coordination of Lum1 and FPB at 1 and 3 cm, and only Lum1 activity at 2 cm. Synergy 2 was a pattern of increased muscle activity with the opening–closing phase, showing intermuscular coordination of Lum1 and FPB at 1 and 3 cm, and only Lum1 activity at 2 cm.

Synergy 2 showed increased muscle activity during the chopstick closing phase, indicating intermuscular coordination between the FPB and APB. Synergies 3, 4, and 5 showed patterns in which the muscle activity increased during the closing phase. Synergy 3 showed the activity of Lum2 alone at 1 cm and intermuscular coordination between Lum2 and TDI at 2 and 3 cm. Synergy 4 showed FDS and TDS activities at 1 and 2 cm, and intermuscular coordination of Lum1 and FDS at 3 cm. Synergy 5 showed only TDI activity.

The non-dominant hand did not show intermuscular coordination between Lum1 and Lum2. The non-dominant hand exhibited intermuscular coordination between the intrinsic and extrinsic muscles of the hand, whereas the dominant hand did not. The non-dominant hand showed intermuscular coordination of the APB and FPB, whereas the dominant hand did not. The dominant hand showed similar synergies regardless of the object size, whereas the non-dominant hand had synergies that varied with different-sized objects. No similarity was observed between the four muscle synergies of the dominant hand and the five muscle synergies of the non-dominant hand.

## 4 Discussion

The present study revealed significant activity differences between dominant and non-dominant hands in 2 of the 11 muscles studied: TDI and ECR. However, no significant differences were observed in the remaining nine muscles. Muscle synergy analysis during chopstick manipulation revealed four synergies in the dominant hand and five in the non-dominant hand. Muscle synergies revealed coordination of hand-specific muscles in both dominant and non-dominant hands; however, the combination of coordinated muscles differed between dominant and non-dominant hands.

### 4.1 Muscle activity

The results of a previous study using motion capture analysis reported that proximal chopstick movements were greater in the non-dominant hand than in the dominant hand (Kamitani et al., 2017). This finding suggests that greater displacement of the proximal chopstick in the non-dominant hand might have led to increased TDI activity, as an additional force was required to stabilize the chopstick with the ring finger.

Previous studies have shown that ECR contributes significantly to wrist stabilization (Ricci et al., 2015). This stabilization is crucial for generating grip strength and performing fine motor tasks with the fingers. It is plausible that the activity of the non-dominant ECR was greater than that of the dominant side in order to maintain wrist stability during the task.

Researchers have reported that unskilled players tend to exert excessive force on their fingers when playing a piano, resulting in greater forearm muscle activity than that in skilled players (Niijima et al., 2021). However, in a study comparing muscle activity between the dominant and non-dominant hands during dart throwing, no significant differences were observed between the sides (Kuhtz-Buschbeck and Keller, 2019). Thus, tasks requiring complex finger movements affect muscle activity based on proficiency, whereas tasks involving simple movements do not show such an effect on muscle activity. In the present study, the analysis interval was from the opening to closing of the chopsticks, and it is possible that this task was not demanding enough to significantly influence muscle activity.

### 4.2 Muscle synergy

The synergies identified in the dominant hand were the intermuscular coordination of the intrinsic muscles, with synergy 1 being the movement of the index and middle fingers, synergy 2 being the movement of the thumb, and synergy 3 being the coordinated movement of the thumb and index fingers. A previous study reported that the index and middle fingers are used to manipulate distal chopsticks, whereas the thumb is used to hold the chopsticks down (Hsu and Wu, 1991). Thus, the extracted synergy patterns are consistent with the expected motor tasks during chopstick manipulation. Here, in the non-dominant hand, intermuscular coordination between the intrinsic and extrinsic muscles was observed. Extrinsic muscles are important for movements that require large forces (Long et al., 1970). Although opening and closing chopsticks do not require a large force, intermuscular coordination between the intrinsic and extrinsic muscles was observed only in the non-dominant hand, suggesting that extra intermuscular coordination may be involved in chopstick manipulation of the non-dominant hand. Furthermore, Lum1 and Lum2 showed intermuscular coordination in the dominant hand but not in the non-dominant hand. Although the lumbrical muscles are important for accurate object manipulation (Wang et al., 2014), intermuscular coordination was observed only in the dominant hand in this study. These muscles have numerous intrinsic receptors and contribute to fine motor performance (Wang et al., 2014), suggesting the importance of intermuscular coordination in skilled chopstick manipulation. This involves not only the movement itself but also the integration of motor and sensory functions.

The difference in intermuscular coordination between the dominant and non-dominant hands may be due to differences in task proficiency. In a previous study comparing muscle synergy between unskilled and skilled Kyudo players (Kyudo is a Japanese sport similar to archery), different muscle synergies were observed, indicating that different levels of task proficiency affect intermuscular coordination (Matsunaga et al., 2017). The results of the present study are consistent with these findings, suggesting that the intermuscular coordination between the dominant and non-dominant hands is different because chopstick manipulation with the non-dominant hand is an unskilled movement. Furthermore, a previous study on motor learning has demonstrated that using the non-dominant hand results in greater activation of brain regions involved in visual processing and motor control than the dominant hand (Kirby et al., 2019). This finding suggests that motor control with the non-dominant hand requires enhanced neural processing to regulate force output and integrate finger and wrist movements. Consequently, this increased neural demand may explain the differences in intermuscular coordination observed between the dominant and non-dominant hands.

## 5 Limitations of this study

This study was conducted with healthy participants; however, it remains unclear whether similar results would be observed in elderly individuals, patients with stroke, or those with impairments in fine motor skills. This is because, in fine motor tasks, training effectiveness is influenced not only by motor function but also by cognitive and sensory function (Fauth et al., 2017). Therefore, further analysis of chopstick manipulation in these populations is needed. The flexor digitorum profundus plays a crucial role in fine-motor tasks (Valenzuela et al., 2024). However, the role of the deep flexor digitorum profundus muscles, located deeper than the superficial flexor muscles, remains unknown, owing to the use of noninvasive surface EMG to avoid interference with manipulation. Moreover, the influence of the deep flexor digitorum on the synergistic effect cannot be assessed. Additionally, muscle synergy analysis between dominant and non-dominant hands was conducted among different individuals because of participant recruitment constraints. Therefore, the effects of individual variability could not be eliminated. A more detailed analysis would require a comparison of dominant and non-dominant muscle synergies in the same participants. However, one advantage of muscle synergy analysis is its ability to extract characteristic muscle activity patterns rather than individual differences. Therefore, the results are more likely to reflect the influence of hand dominance rather than individual differences (Ferrante et al., 2016).

### 5.1 Future prospects

The results of this study suggest that focusing on intermuscular coordination may be an important element in rehabilitation strategies to enable patients with stroke to use chopsticks with their non-dominant hand through dominant hand exchange. In the future, more specific rehabilitation approaches will emerge from examining the direct relationship between improved intermuscular coordination and enhanced chopstick use through functional outcomes.

## 6 Conclusion

The present study aimed to clarify the differences in muscle activity and intermuscular coordination between the dominant and non-dominant hand during chopstick manipulation and to inform rehabilitation strategies. We proposed two research hypotheses: (1) muscle activity during chopstick manipulation in the non-dominant hand is greater than that in the dominant hand, and (2) intermuscular coordination differs between the hands. We found significant differences in activity between the dominant and non-dominant hands in two muscles, TDI and ECR. However, we observed no significant differences were observed in the remaining nine muscles, partially supporting our hypothesis. Our findings also revealed differences in how muscles interacted with each between the dominant and non-dominant hands. In particular, the first and second lumbrical muscles cooperated in the dominant hand, whereas they did not two in the non-dominant hand. These findings suggest that intermuscular coordination between the first and second lumbrical muscles is important for developing rehabilitation strategies to improve the use of chopsticks in the non-dominant hand. An effective approach to improving the interaction between these muscles may involve repetitive training of flexion-extension movements of the interphalangeal joints of the index and middle fingers. Such targeted training could enhance intermuscular coordination in the non-dominant hand and ultimately facilitate smoother chopstick manipulation.

## Data Availability

The original contributions presented in the study are included in the article/supplementary material, further inquiries can be directed to the corresponding author.
